# Contrasting Patterns and Drivers of Soil Fungal Communities between Two Ecosystems Divided by the Treeline

**DOI:** 10.3390/microorganisms9112280

**Published:** 2021-11-02

**Authors:** Xueying Wang, Guixiang Li, Yuxin Zhang, Keming Ma

**Affiliations:** 1 State Key Laboratory of Urban and Regional Ecology, Research Center for Eco-Environmental Sciences, Chinese Academy of Sciences, Beijing 100085, China; wangxueying191@mails.ucas.ac.cn (X.W.); yxzhang@rcees.ac.cn (Y.Z.); 2 University of Chinese Academy of Sciences, Beijing 100049, China; 3 Weifang Academy of Agricultural Sciences, Weifang 261061, China; guixiangli2010@163.com

**Keywords:** treeline, soil fungi, Illumina Miseq, community composition, ecological network

## Abstract

The treeline is a sensitive region of the terrestrial ecosystem responding to climate change. However, studies on the composition and formation mechanisms of soil fungal communities across the treeline are still lacking. In this study, we investigated the patterns of soil fungal community composition and interactions among functional guilds above and below the treeline using Illumina high-throughput sequencing and ecological network analysis. The results showed that there were significant differences in the soil environment and soil fungal community composition between the two ecosystems above and below the treeline. At the local scale of this study, geographic distance and environmental factors affected the composition of the soil fungal community. Soil temperature was an important environmental predictor of soil fungal community composition. Species in soil fungal communities in the subalpine meadow were more closely related to each other compared to those in the montane forest. Furthermore, the soil fungal community in montane forest was more stable. Our findings contribute to a better understanding of how mountain ecological functions respond to global climate change.

## 1. Introduction

Mountains are hotspots of biodiversity and create significant environment divergence over short distances [1], providing natural space-for-time settings for studies of community in situ responses to long-term climate change [2]. A striking manifestations of rapid environmental change is the transition from continuous closed forest canopy to treeless alpine tundra, that is, the formation of treelines [3]. It is an important ecological transition zone of the alpine vertical vegetation zone and a sensitive region of the terrestrial ecosystem responding to climate change [4]. Several studies have explored the formation mechanism of the treeline [5], its litter decomposition [6], and the soil carbon and nitrogen cycle [7]; however, there are relatively few studies on soil microorganisms at the treeline, and these are limited to the degradation activity of microorganisms [8] and soil bacterial diversity [9]. The composition pattern and the formation mechanism of the soil fungal community remain understudied.

Fungi are an important component of soil microorganisms and have important ecological functions; for example, they are involved in plant litter decomposition and nutrient cycling [10], and they form symbionts with higher plants [11] and act as pathogens [12]. Their decomposition function is a key factor for carbon storage and release in soil; therefore, systemic changes in the function of soil fungal community in mountain ecosystems could have global consequences [13]. Considering the response of fungal community composition to climate change, comparative studies of multiple climatic zones on a large scale [14] or artificial warming experiments on a local scale [15] are commonly being conducted at present. However, a unified conclusion has not been reached on how climate change affects the synergistic changes in vegetation, soil, and fungal communities in natural ecosystems at local scales.

Based on high-throughput sequencing, our ability to know the richness and composition of microbial communities has been greatly improved. Before, researchers were often limited by the inability to relate operational taxonomic units (OTUs) to their ecological meaning [16]. The FUNGuild database parses a group of species that use the same class of environmental resources in a similar way into a guild [17]. The use of guilds allows for comparative studies among different communities whether the species are related or unrelated [18]. Therefore, soil fungi were parsed into three trophic modes: saprotrophs, symbiotrophs, and pathotrophs, as well as 12 detailed functional guilds according to the trophic strategies of fungal species [19]. As each functional group may have different responses to environmental variables, and there are interactions among each group [20], it is helpful to fully understand the changes in the composition and function of the fungal community by dividing the whole community into different groups based on trophic strategies.

In recent years, network analysis-based methods have been used to study the symbiotic patterns of microorganisms in forest soils [21]. Ecological network analysis is a method based on mathematical model to study the interaction relationships within ecosystems [22]. With the development of this method, combined with high-throughput sequencing data, we can explore the changes of the potential interaction relationships in the fungal communities above and below the treeline [23,24].

In this study, we collected soil samples from the montane forest and subalpine meadow above and below the treeline of Dongling Mountain, respectively. Using Illumina high-throughput sequencing technology combined with the ecological network method, we analysed the composition pattern of soil fungal communities and the interactions among different guilds. Particularly, this study aimed to explore: (1) the specific differences in the composition of soil fungal communities and their formation mechanisms in different ecosystems above and below the treeline; and (2) the changes that might have occurred in the interaction among soil fungal functional guilds after crossing the treeline. Based on previous studies, we hypothesized that (1) different soil fungal functional guilds would have their own special distribution driving mechanism; and (2) the interaction among soil fungal functional guilds becomes closer and the community stability becomes worse after crossing the treeline.

## 2. Materials and Methods

### 2.1. Study Site and Soil Sampling

The study site was located on Dongling Mountain (40°00′–40°03′ N, 115°26′–115°30′ E), approximately 100 km northwest of Beijing, China. This area is characterized by a warm temperate continental monsoon climate, with a mean annual air temperature of 6.5 °C and mean annual precipitation of 600 mm. The soil types are mainly mountain brown soil, subalpine meadow soil, and cinnamon soil. The parent material of soil formation mainly includes granite, sandstone, conglomerate, and andesite. The natural vegetation of the region is generally divided into two types by elevations: montane forest (1000–1900 m) and subalpine meadow (1700–2300 m). The treeline is located about 1770 m.

**Figure 1:**
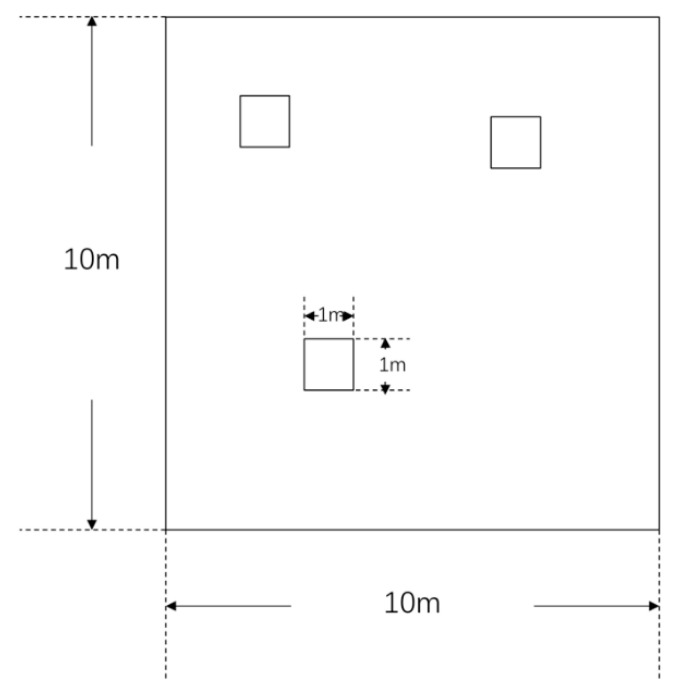
Soil samples were collected from three independent quadrats (1 m × 1 m) within each plot (10 m × 10 m).

Soil samples were collected in August. In the montane forest, we set 7 transects from 1250 to 1770 m with a width of 10 m. We set plots at the upper, middle and lower slope positions of the 7 transects, with a total of 21 plots (10 m × 10 m, further than 50 m apart). In the subalpine meadow, we set 21 plots (10 m × 10 m, further than 50 m apart) from 1770–2280 m. Within each plot, we selected three independent quadrats (1 m × 1 m) and recorded the abundance for all herb species (Figure 1). Six soil samples were collected from three quadrats using an antiseptic foil sampler at a depth of 10 cm and pooled to yield a composite sample for each plot. The fresh soil samples were thoroughly homogenized and passed through a 2 mm antiseptic sieve. Subsamples were air-dried for the physical and chemical analyses, and the others were kept at −80 °C until DNA extraction.

### 2.2. Measurement of Soil Parameters

Soil pH was determined at a ratio of 1:2.5 (soil to water, *w*/*v*). Soil moisture (SM) was measured gravimetrically. Total nitrogen (TN) and total carbon (TC) were measured by direct combustion using an element analyser (Vario EL III, Langenselbold, Germany). Available phosphorus (AP) was measured using the Mo-Sb anti-spectrophotometry method after extraction using sodium bicarbonate solution. Available nitrogen (AN) was measured through the alkaline hydrolysis diffusion method [25]. After acid dissolution, total phosphorus (TP) was measured using Mo-Sb colorimetry method. Soil texture was analysed using a Mastersizer 2000 Laser Diffraction Particle Analyzer (Malvern Instruments, Malvern, UK). The soil particle size was partitioned into clay (0–2 μm), silt (2–50 μm), and sand (50–2000 μm) according to the classification system of the US Department of Agriculture. We used iButton (1922L, supported by Maxim Integrated, San Jose, CA, USA), set to record temperatures automatically each hour. The thermometers were deployed in all 42 plots at the same time and were buried at a depth of 10 cm into the ground so as to decrease the occurrence of extreme values and skewed data. The daily average soil temperature during the sampling period was calculated using the data recorded by the button thermometer.

### 2.3. DNA Extraction and Sequencing

Following the manufacturer’s instructions, total DNA was extracted from 0.25 g freeze-dried soil samples using the MOBIO Power Soil DNA extraction kit (MO Bio Laboratories, Carlsbad, CA, USA). The concentration and quality of the DNA were evaluated using NanoDrop spectrophotometer (Thermo Fisher Scientific, Wilmington, DE, USA). The forward and reverse primers used for PCR amplification were ITS3F: GCATCGATGAAGAACGCAGC and ITS4R: TCCTCCGCTTATTGATATG [26]. Each amplification was carried out in a 25-μL reaction mixture, containing 4 μL 5× FastPfu buffer, 2 μL 2.5 mmol/L dNTPs, 0.4 μL of each primer (5 mmol/L), 0.4 μL FastPfu polymerase (TransGen, Beijing, China), and 10 ng template DNA. The following cycling parameters were used: 95 °C for 2 min; 30 cycles at 95 °C for 30 s, 55 °C for 30 s, 72 °C for 45 s; followed by 72 °C for 10 min. The reactions were performed in triplicate for each sample to minimize the random PCR bias and were then pooled together. The PCR products were purified using the AxyPrepDNA Gel Extraction Kit (AXYGEN, Union City, CA, USA). Equal concentrated amplicons were paired-end sequenced (PE 2 × 300) on Illumina MiSeq platform (Illumina, San Diego, CA, USA).

Quality trimming was performed using Trimmomatic [27]. Pairs of reads were merged by FLASH [28] according to the overlap. Primary qualified and merged sequences were analysed in QIIME v.1.8.0 software package [29]. Sequences containing ambiguous bases that could not be assigned to a sample using the barcodes, and homopolymers with >8 bases, <200 bp in length, and with an average quality score < 30 were removed. After quality filtering, the chimeras were checked using UCHIME [30] against the “RDP Gold” database and clustered into OTUs based on 97% similarity with the USEARCH option [31]. Taxonomy was identified for each OTU using the RDP classifier trained on the UNITE database [32]. All of the sequence data have been submitted to the GenBank Sequence Read Archives (http://www.ncbi.nlm.nih.gov, accessed on 29 September 2021) under BioProject ID PRJNA767343. Functions were predicted based on fungal taxa using the FUNGuild database (http://www.stbates.org/guilds/app.php, accessed on 21 October 2020) and only Probable and Highly Probable assignments were accepted. 

### 2.4. Statistical Analysis

We calculated OTU richness and Shannon index for the measured fungal communities. To compare the fungal richness, soil and plant parameters between montane forest and subalpine meadow, a *t*-test was used. Community compositional dissimilarities were estimated based on the Bray–Curtis distance of the OTU abundance table. Principal coordinates analysis (PCoA) was performed to examine the fungal community variation and Adonis test (“vegan” package in R) was used to test the significance.

All environmental variables were standardised at a mean of 0 and a standard deviation of 1. Matrices of environmental variables and geographic distances were generated based on Euclidean distances. Mantel tests (mantel test, “vegan” package in R) were used to test the correlations among community composition, and environmental and geographic distances. After controlling the correlation between environmental factors, the effects of various environmental factors on soil fungal community composition were further explored through Partial Mantel tests (Mantel Partial test, “vegan” package in R). Spearman rank correlation analysis was used to analyse the relationship between the relative abundances of predicted functional guilds of fungi and environmental variables.

Molecular ecological networks of soil fungi for montane forests and subalpine meadows were constructed using sequencing data with OTUs whose relative abundances exceeded 0.1% of the total fungal sequences. The number of species was corrected before the network was established. The construction of the network and the acquisition of network property parameters were completed on the online analysis pipeline: Molecular Ecological Network Analyses Pipeline (http://ieg4.rccc.ou.edu/mena/, accessed on 8 April 2021) and visualised using Cytoscape 3.8.2 software. Connectivity refers to the connectivity strength between one node and other connected nodes; geodesic distance is the shortest distance between two nodes; clustering coefficient is a coefficient of aggregation tightness between a node and its connected nodes; modularity refers to the characteristics of modules in molecular ecological networks. A network is divided into multiple modules, and a single module is considered to be a functional unit in the ecosystem [23]. Maslov–Sneppen method was used to reconnect the nodes at different locations in the original network without changing the number of original network nodes and connections, and 100 random networks were constructed [22]. Then the differences between molecular ecological networks and random networks were compared. *t*-test was used to analyse the differences of fungal network structure between different regions.

## 3. Results

### 3.1. Plant Parameters and Soil Properties

There were significant differences in the number of herbaceous species, but not in the number of plant species and the thickness of litter between montane forest and subalpine meadow (Table 1). There were many differences in soil properties between the two habitats. Compared with the montane forest, soil temperature significantly decreased, and SM, TN, TC, TP, AN, and AP significantly increased in the subalpine meadow. These results indicate that subalpine meadow soil was relatively richer in nutrients than that of the montane forest.

### 3.2. Fungal Community Diversity and Composition

Across all the 42 soil samples, 5091 OTUs were identified (631,332 sequences) with a range from 9973 to 19,124 in each sample (Figure S1 in Supporting Information). Alpha diversity analysis of the communities showed that the richness and Shannon index of the subalpine meadow fungal community at the OTU level were higher than those of the montane forest fungal community, although there was no significant difference (Table 1).

**Figure 2:**
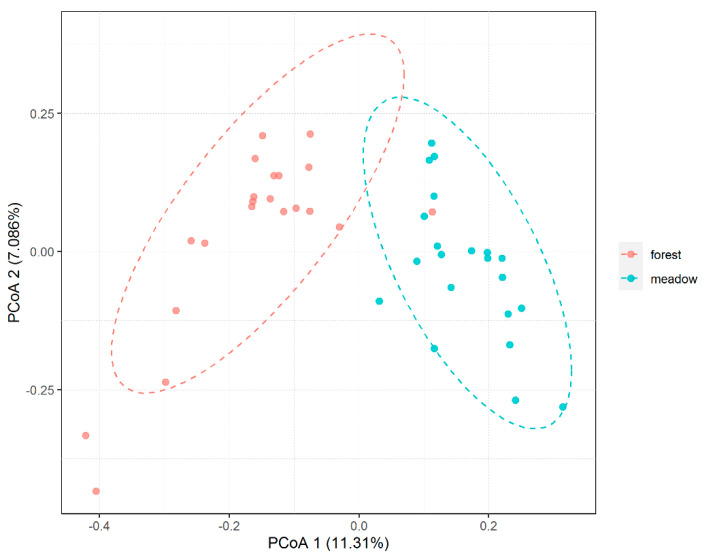
Principal coordinates analysis (PCoA) based on Bray−Curtis distance of fungal communities between forest (below the treeline) and meadow (above the treeline). Ellipses were added according to the sample grouping of experimental design and specified a confidence of 0.95 when fitting the ellipse radius.

PCoA based on Bray–Curtis distance showed that soil fungal communities in the same ecosystem were closely distributed and were significantly separated from those in the other ecosystem (Figure 2). The results of the PERMANOVA test showed that the fungal communities in the two ecosystems were significantly different (Adonis *F*_1,40_ = 4.0896, *R*^2^ = 0.093, *p* = 0.001). There were 1720 identical OTUs in the two ecosystems; in addition, 1938 OTUs were unique to the forest and 1433 OTUs only occurred in the meadow (Figure S2 in Supporting Information).

**Figure 3:**
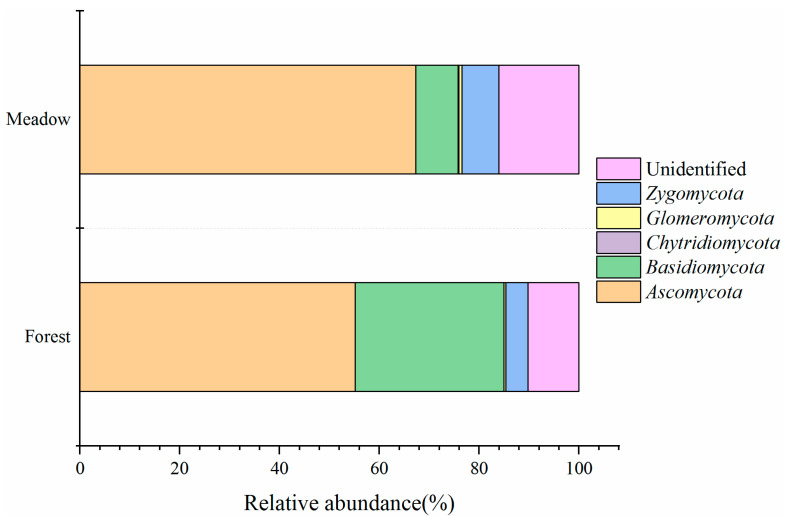
Comparison of fungal community composition between forest (below the treeline) and meadow (above the treeline) at the phylum level.

The relative abundance of the taxonomic composition of the fungal community at the phylum level is shown in Figure 3. A total of five fungal phyla were detected, and the dominant phyla in both the ecosystems were *Ascomycota*, *Basidiomycota*, and *Zygomycota*. The relative abundance of *Ascomycota*, *Basidiomycota*, and *Zygomycota* in the forest were 55.1%, 29.7%, and 5.6%, respectively, while those in the meadow were 67.3%, 8.5%, and 7.4%, respectively. These results indicate that the abundance of *Ascomycota* was higher, while that of *Basidiomycota* was significantly lower in the meadow compared to that in the forest (*p* < 0.05, Table S1).

**Figure 4:**
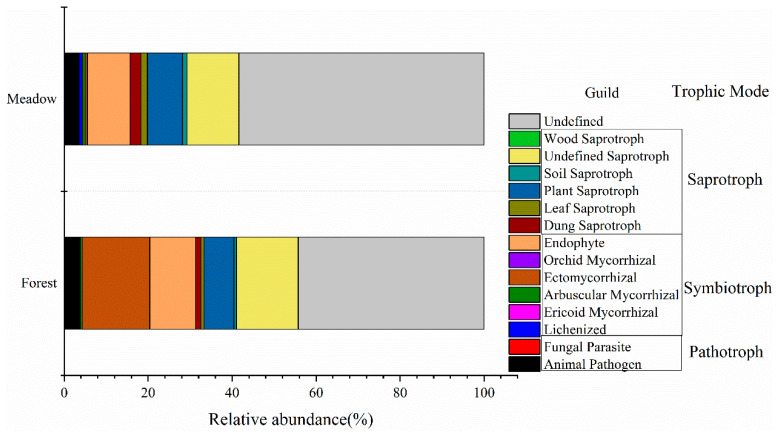
Comparison of the composition of fungal functional guilds inferred by FUNGuild between forest (below the treeline) and meadow (above the treeline).

In the identified trophic modes of fungi (Figure 4), the relative abundance of pathotrophs, symbiotrophs, and saprotrophs in the forest was 24.0%, 29.2%, and 4.0%, respectively. The relative abundance of the three types in the meadow was 26.0%, 12.1%, and 3.6%, respectively, indicating that the symbiotrophs were significantly less abundant in the meadow (*p* < 0.05, Table S1).

### 3.3. Relationship of Biotic and Abiotic Factors with Fungal Community Structure

Mantel test confirmed that both geographic distance and environmental distance had significant effects on soil fungal community composition (Table 2). After controlling for the correlation between environmental factors, the effects of various environmental factors on the composition of soil fungal communities were further explored through Partial Mantel tests (Table 3). The results showed that soil temperature had a significant effect on the composition of the soil fungal community.

The relative abundance of different functional guilds of fungi was correlated with various environmental factors (Table S2 and Figure S3). Among them, leaf saprotrophs were the most abundant saprotrophs, and their presence significantly positively correlated with the herbaceous richness. In the case of symbiotrophs, ectomycorrhiza were the most abundant and significantly negatively correlated with the herbaceous richness and significantly positively correlated with the soil temperature. Ericoid mycorrhiza, which accounted for a relatively small proportion of symbiotrophs, were significantly negatively correlated with the soil nutrients. No environmental factors were found to be significantly correlated with the relative abundance of pathotrophs.

### 3.4. Fungal Ecological Network Analysis above and below the Treeline

The fungal molecular ecological network analysis revealed that the parameters of the network established with the same correlation threshold (0.60) were different between the forest and the meadow (Table S3). The average geodesic distance, average clustering coefficient, and modularity of the molecular ecological network presented larger values than those of the random network, and the network constructed conformed to features of the network, such as scale-free, small world, and modular.

The average connectivity of the soil fungal community network in the meadow was significantly higher than that in the forest (*p* < 0.05), and the average geodesic distance and modularity of the network were significantly lower than that in the forest (*p* < 0.05). The number of positive correlation lines in forest and meadow networks was 88 and 78, respectively, and accounted for 72% and 66% of the corresponding total number of lines.

**Figure 5:**
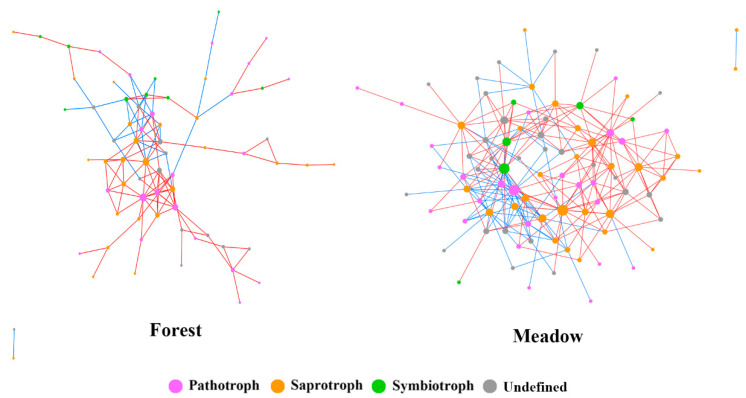
Ecological networks of soil fungal communities between forest (below the treeline) and meadow (above the treeline). The red line indicates a positive correlation, and the blue line indicates a negative correlation. The colours of the nodes represent different trophic modes. The size of each node is proportional to the connectivity.

We divided all nodes in the two networks into trophic modes (Figure 5). In the forest fungal network, the number of nodes classified into undefined fungi, pathotrophs, symbiotrophs, and saprotrophs was 14, 22, 9, and 25, accounting for 20%, 32%, 13%, and 36% of the total nodes, respectively. In the meadow fungal network, the number of nodes divided into undefined fungi, pathotrophs, symbiotrophs, and saprotrophs was 29, 26, 6, and 30, accounting for 32%, 28%, 7%, and 33% of the total nodes, respectively. Although a large proportion of fungi could not be identified and divided into definite trophic modes, they were indeed key nodes in the network. In addition, saprotrophs accounted for the largest proportion of nodes in both networks.

## 4. Discussion

In this study, soil temperature was a significant driver of soil fungal community composition. Plant richness did not have a direct and significant effect on soil fungal community composition; however, with a combination of vegetation composition and elevation, different soil environments were formed above and below the treeline. In previous studies, the main drivers of the differences between fungal communities were temperature and the associated long-term development of plant communities and their soil environment [33]. Different vegetation has specific ecosystem functions and traits, and input organic and inorganic nutrients to the soil in the form of litter decomposition and rhizodeposition [34]. Therefore, the broadleaved deciduous forest below the treeline and the meadow above the treeline inevitably input different types of nutrients into the soil in Dongling Mountain. In addition, the low temperature of the subalpine meadow hinders the decomposition of fine roots and leaf litter, resulting in a high content of organic matter in the topsoil [35]. This increases the available resources to fungi. While soil nutrients have a higher turnover rate due to higher temperature, the available nutrients in surface soil decrease in the montane forest.

When exploring the influence of environmental factors on the fungal community, the groups with higher relative abundance will contribute more to the composition pattern of the entire fungal community [36]. However, among different fungal groups (at a finer taxonomic scale), environmental predictors tend not to be similar [37]. In this study, we investigated the distribution of fungi under the system of functional prediction. Ectomycorrhiza, the most abundant symbiotrophs, often form mycorrhiza with woody plants of Fagaceae [38], which are widely distributed in the montane forest; therefore, the distribution of Ectomycorrhiza above and below the treeline showed obvious difference. In addition, ericoid mycorrhiza which account for a relatively small proportion of symbiotrophs, their presence significantly negatively correlated with the soil nutrients. They may play a key role in the change of the whole community structure as a specialist in the soil fungal community [39]. The difference in the distribution of symbiotrophs is the ecological consequence of forest becoming meadow and woody disappearing, which is embodied in the response of mycorrhizal to plant community in different ecosystems [40]. The change of mycorrhizal is an important part of the change of fungal community composition. These results clearly show that fungi with different trophic modes have different environmental preferences and responses to environmental filtering, thus forming different patterns of fungal community composition.

Moreover, the scale dependence of the formation mechanisms of microbial spatial structure has been demonstrated in previous studies, such as dispersal limitation and environmental filtering, whose relative importance varies with geographic scale [41]. In this study, both geographical distance and environmental factors had significant effects on soil fungal community composition, which supported the conclusion that dispersal limitation and environmental filtering together contribute to the formation of soil fungal community patterns at a local scale. Boraks et al. found that geographical distance best explained the turnover of underground fungal communities at a small scale [41]. A study in Southern California showed that soil fungal community composition patterns formed through deterministic environmental filtering, with no significant effect of dispersal limitation at a regional scale [42]. In general, fungal community composition is strongly influenced by climatic factors at large scales, in which environmental filtering plays a dominant role, while dispersal limitation plays a more significant role at smaller scales. In conclusion, the dominant process of microbial spatial structure formation largely depends on the scale of the sampling location.

Co-occurrence network analysis can help us to explore the interaction between fungal functional guilds, which can reflect ecological processes such as cooperation, competition, environmental filtering, and historical effects [43]. The results of network analysis showed that the soil fungal community composition pattern in the montane forest and subalpine meadow was non-random co-occurrence. The difference on average geodesic distance and average connectivity indicated that the soil fungal network structure of meadow soil was more compact and the interaction between species more intense compared with the forest soil fungal network. When the disturbance occurs in the external environment, the structure will quickly transmit the disturbance to the whole network in the closely connected network [44]. In contrast, modular organization can buffer communities against secondary extinctions following disturbance and increase overall network stability [45]. According to the co-occurrence network theory [46], the forest soil fungal community with higher modularity has stronger resistance and higher stability compared with the soil fungal community in the subalpine meadow. Positive correlations were dominant in both networks, suggesting that cooperation may play an important role in the formation of species co-occurrence patterns [47]. Saprotrophs accounted for the largest proportion of nodes in both networks, suggesting that they played a vital role in the fungal community composition pattern. In general, the interaction of species in the soil fungal community was closer after crossing the treeline, but the overall structural stability decreased. Treeline advancement in alpine areas has been verified in previous studies [5]. Our results showed that after crossing the treeline, the stability of soil fungal community decreased and soil organic matter began to accumulate, indicating that treeline advancement in alpine areas may weaken soil decomposition function and slow down nutrient cycling.

## 5. Conclusions

The results showed that there were significant differences in soil environment and soil fungal community composition between the two ecosystems above and below the treeline. At the local scale of this study, geographic distance and environmental factors affected the composition of the soil fungal community. Soil temperature was the most important environmental predictor of soil fungal community composition; however, different functional guilds had different responses to the same environmental factors. Network analysis showed that saprotrophs are the key functional groups in both ecosystems. Species in soil fungal communities in the subalpine meadow were more closely related to each other, whereas soil fungal community in the montane forest was more stable. It can be predicted that the upshift of treelines caused by global warming will lead to significant changes in soil environment and soil microbial community, and the results of this study contribute to a better understanding of how mountain ecological functions respond to global climate change.

## Figures and Tables

**Table 1:** Summary of biotic and abiotic parameters of montane forest and subalpine meadow and significance of differences based on *t*-tests.

	Forest	Meadow	*t*-Tests (*p* Value)
Plant parameters			
Plant richness	32.19 ± 7.55	30.48 ± 5.96	0.419
Herb richness	24.43 ± 7.85	30.48 ± 5.96	**0.008**
Litter thickness (cm)	1.37 ± 0.93	1.13 ± 0.66	0.345
Microbial parameters			
Observed richness	493.14 ± 148.45	497.48 ± 170.79	0.931
Shannon index	3.8 ± 0.85	4.12 ± 0.73	0.207
Soil parameters			
Temperature (°C)	17.97 ± 1.17	15.18 ± 1.69	**<** **0.001**
SM (%)	0.39 ± 0.11	0.5 ± 0.15	**0.011**
Bulk density (g/cm^3^)	82.45 ± 14.3	73.89 ± 8.26	**0.022**
pH	6.28 ± 0.6	6.34 ± 0.26	0.679
Clay (%)	17.14 ± 5.01	15.12 ± 4.91	0.196
Silt (%)	60.59 ± 3.1	67.15 ± 4.74	**<** **0.001**
Sand (%)	22.27 ± 5.46	17.73 ± 4.87	**0.007**
TN (%)	0.33 ± 0.06	0.51 ± 0.11	**<** **0.001**
TC (%)	4.89 ± 0.92	7.15 ± 1.7	**<** **0.001**
C:N molar ratio	17.12 ± 1.31	16.29 ± 0.68	**0.014**
AP (mg/kg)	3.92 ± 0.7	6.76 ± 2.48	**<** **0.001**
AN (mg/kg)	370 ± 62.99	585.99 ± 136.39	**<** **0.001**
TP (mg/kg)	657.51 ± 115.61	952.39 ± 207.9	**<** **0.001**

*p* < 0.05 is shown in bold. SM, soil moisture; TN, total nitrogen; TC, total carbon; C:N ratio, ratio of total carbon and total nitrogen; AP, available phosphorus; AN, available nitrogen; TP, total phosphorus.

**Table 2:** Mantel test for the correlation of fungal community dissimilarity with environmental distance and geographic distance.

Explanatory Distance	*R*	*p*
Geographic distance	0.1692	**0.001**
Environmental distance	0.1467	**0.019**

*p* < 0.05 is shown in bold.

**Table 3:** The relationships of fungal community structure to environment variables revealed by Partial Mantel tests.

Variables	*R*	*p*
Herb richness	0.001	0.478
Plant richness	−0.0633	0.745
Temperature	**0.1487**	**0.025**
SM	−0.029	0.644
Litter thickness	−0.0951	0.864
BD	0.0876	0.174
pH	0.0815	0.227
Clay	0.0186	0.354
Silt	0.018	0.384
Sand	−0.1241	0.957
TN	0.0414	0.304
TC	−0.0214	0.592
C:N ratio	0.106	0.151
AP	0.0548	0.281
AN	0.0246	0.369
TP	0.0462	0.264

*p* < 0.05 is shown in bold. SM, soil moisture; BD, soil bulk density; TN, total nitrogen; TC, total carbon; C:N ratio, ratio of total carbon and total nitrogen; AP, available phosphorus; AN, available nitrogen; TP, total phosphorus.

## Data Availability

All of the sequence data have been submitted to the GenBank Sequence Read Archives (http://www.ncbi.nlm.nih.gov, accessed on 29 September 2021) and available in SRA (sequence read archive) under accession number PRJNA767343.

## Supplementary Materials

The following are available online at https://www.mdpi.com/article/10.3390/microorganisms9112280/s1. Table S1: Summary of fungal community composition between forest and meadow and significance of differences based on *t*-tests; Table S2: Spearman’s correlation between relative abundance of functional guilds and environmental variables; Table S3: Molecular ecological network and random network parameters of forest and meadow soil fungal communities; Figure S1: Rarefaction curves of high-throughput sequencing data of the samples; Figure S2: Venn diagram showing numbers of unique and shared fungal OTUs between forest and meadow; Figure S3: Spearman’s correlation between relative abundance of functional guilds and environmental variables.

## References

[1] Martin P.H., Bellingham P.J. (2016). Towards integrated ecological research in tropical montane cloud forests. J. Trop. Ecol..

[2] Malhi Y., Silman M., Salinas N., Bush M., Meir P., Saatchi S. (2010). Introduction: Elevation gradients in the tropics: Laboratories for ecosystem ecology and global change research. Glob. Chang. Biol..

[3] Korner C., Paulsen J. (2004). A world-wide study of high altitude treeline temperatures. J. Biogeogr..

[4] Mayor J.R., Sanders N.J., Classen A.T., Bardgett R.D., Clement J.C., Fajardo A., Lavorel S., Sundqvist M.K., Bahn M., Chisholm C. (2017). Elevation alters ecosystem properties across temperate treelines globally. Nature.

[5] Harsch M.A., Hulme P.E., McGlone M.S., Duncan R.P. (2009). Are treelines advancing? A global meta-analysis of treeline response to climate warming. Ecol. Lett..

[6] Zhou Y., Wang L.F., Chen Y.M., Zhang J., Liu Y. (2020). Litter stoichiometric traits have stronger impact on humification than environment conditions in an alpine treeline ecotone. Plant Soil.

[7] Kammer A., Hagedorn F., Shevchenko I., Leifeld J., Guggenberger G., Goryacheva T., Rigling A., Moiseev P. (2009). Treeline shifts in the Ural mountains affect soil organic matter dynamics. Glob. Chang. Biol..

[8] Withington C.L., Sanford R.L. (2007). Decomposition rates of buried substrates increase with altitude in the forest-alpine tundra ecotone. Soil Biol. Biochem..

[9] Li G.X., Xu G.R., Shen C.C., Tang Y., Zhang Y.X., Ma K.M. (2016). Contrasting elevational diversity patterns for soil bacteria between two ecosystems divided by the treeline. Sci. China-Life Sci..

[10] Clipson N., Otte M., Landy E. (2006). Fungi in Biogeochemical Cycles: Biogeochemical Roles of Fungi in Marine and Estuarine Habitats.

[11] Yang Y., Dou Y.X., Huang Y.M., An S.S. (2017). Links between Soil Fungal Diversity and Plant and Soil Properties on the Loess Plateau. Front. Microbiol..

[12] Geml J., Pastor N., Fernandez L., Pacheco S., Semenova T.A., Becerra A.G., Wicaksono C.Y., Nouhra E.R. (2014). Large-scale fungal diversity assessment in the Andean Yungas forests reveals strong community turnover among forest types along an altitudinal gradient. Mol. Ecol..

[13] Looby C.I., Martin P.H. (2020). Diversity and function of soil microbes on montane gradients: The state of knowledge in a changing world. FEMS Microbiol. Ecol..

[14] Mukhtar H., Lin C.M., Wunderlich R.F., Cheng L.C., Ko M.C., Lin Y.P. (2021). Climate and land cover shape the fungal community structure in topsoil. Sci. Total Environ..

[15] Corneo P.E., Pellegrini A., Cappellin L., Gessler C., Pertot I. (2014). Moderate Warming in Microcosm Experiment Does Not Affect Microbial Communities in Temperate Vineyard Soils. Microb. Ecol..

[16] Nguyen N.H., Song Z.W., Bates S.T., Branco S., Tedersoo L., Menke J., Schilling J.S., Kennedy P.G. (2016). FUNGuild: An open annotation tool for parsing fungal community datasets by ecological guild. Fungal Ecol..

[17] Root R.B. (1967). NICHE EXPLOITATION PATTERN OF BLUE-GRAY GNATCATCHER. Ecol. Monogr..

[18] Hawkins C.P., Macmahon J.A. (1989). GUILDS—THE MULTIPLE MEANINGS OF A CONCEPT. Annu. Rev. Entomol..

[19] Tedersoo L., Bahram M., Polme S., Koljalg U., Yorou N.S., Wijesundera R., Ruiz L.V., Vasco-Palacios A.M., Thu P.Q., Suija A. (2014). Global diversity and geography of soil fungi. Science.

[20] Xie F., Ma A.Z., Zhou H.C., Liang Y., Yin J., Ma K., Zhuang X.L., Zhuang G.Q. (2020). Revealing Fungal Communities in Alpine Wetlands through Species Diversity, Functional Diversity and Ecological Network Diversity. Microorganisms.

[21] Toju H., Kishida O., Katayama N., Takagi K. (2016). Networks Depicting the Fine-Scale Co-Occurrences of Fungi in Soil Horizons. PLoS ONE.

[22] Luo F., Yang Y., Zhong J., Gao H., Khan L., Thompson D.K., Zhou J. (2007). Constructing gene co-expression networks and predicting functions of unknown genes by random matrix theory. BMC Bioinform..

[23] Zhou J.Z., Deng Y., Luo F., He Z.L., Tu Q.C., Zhi X.Y. (2010). Functional Molecular Ecological Networks. mBio.

[24] Zhou J.Z., Deng Y., Luo F., He Z.L., Yang Y.F. (2011). Phylogenetic Molecular Ecological Network of Soil Microbial Communities in Response to Elevated CO_2_. mBio.

[25] Cornfield A.H. (1960). Ammonia released on treating soils with N sodium hydroxide as a possible means of predicting the nitrogen-supplying power of soils. Nature.

[26] White T.J., Bruns T.D., Lee S.B., Taylor J.W. (1990). Amplification and direct sequencing of fungal ribosomal RNA Genes for phylogenetics. PCR Protocols.

[27] Bolger A.M., Lohse M., Usadel B. (2014). Trimmomatic: A flexible trimmer for Illumina sequence data. Bioinformatics.

[28] Magoc T., Salzberg S.L. (2011). FLASH: Fast length adjustment of short reads to improve genome assemblies. Bioinformatics.

[29] Caporaso J.G., Kuczynski J., Stombaugh J., Bittinger K., Bushman F.D., Costello E.K., Fierer N., Pena A.G., Goodrich J.K., Gordon J.I. (2010). QIIME allows analysis of high-throughput community sequencing data. Nat. Methods.

[30] Edgar R.C., Haas B.J., Clemente J.C., Quince C., Knight R. (2011). UCHIME improves sensitivity and speed of chimera detection. Bioinformatics.

[31] Edgar R.C. (2010). Search and clustering orders of magnitude faster than BLAST. Bioinformatics.

[32] Abarenkov K., Nilsson R.H., Larsson K.H., Alexander I.J., Eberhardt U., Erland S., Hoiland K., Kjoller R., Larsson E., Pennanen T. (2010). The UNITE database for molecular identification of fungi-recent updates and future perspectives. New Phytol..

[33] Chen L., Xiang W.H., Wu H.L., Ouyang S., Lei P.F., Hu Y.J., Ge T.D., Ye J., Kuzyakov Y. (2019). Contrasting patterns and drivers of soil fungal communities in subtropical deciduous and evergreen broadleaved forests. Appl. Microbiol. Biotechnol..

[34] Mitchell R.J., Hester A.J., Campbell C.D., Chapman S.J., Cameron C.M., Hewison R.L., Potts J.M. (2010). Is vegetation composition or soil chemistry the best predictor of the soil microbial community?. Plant Soil.

[35] Wang D., He N.P., Wang Q., Lu Y.L., Wang Q.F., Xu Z.W., Zhu J.X. (2016). Effects of Temperature and Moisture on Soil Organic Matter Decomposition Along Elevation Gradients on the Changbai Mountains, Northeast China. Pedosphere.

[36] Shen C.C., Gunina A., Luo Y., Wang J.J., He J.Z., Kuzyakov Y., Hemp A., Classen A.T., Ge Y. (2020). Contrasting patterns and drivers of soil bacterial and fungal diversity across a mountain gradient. Environ. Microbiol..

[37] Yeh C.F., Soininen J., Teittinen A., Wang J.J. (2019). Elevational patterns and hierarchical determinants of biodiversity across microbial taxonomic scales. Mol. Ecol..

[38] Gao C., Zhang Y., Shi N.N., Zheng Y., Chen L., Wubet T., Bruelheide H., Both S., Buscot F., Ding Q. (2015). Community assembly of ectomycorrhizal fungi along a subtropical secondary forest succession. New Phytol..

[39] Leopold D.R. (2016). Ericoid fungal diversity: Challenges and opportunities for mycorrhizal research. Fungal Ecol..

[40] Genre A., Lanfranco L., Perotto S., Bonfante P. (2020). Unique and common traits in mycorrhizal symbioses. Nat. Rev. Microbiol..

[41] Boraks A., Plunkett G.M., Doro T.M., Alo F., Sam C., Tuiwawa M., Ticktin T., Amend A.S. (2021). Scale-Dependent Influences of Distance and Vegetation on the Composition of Aboveground and Belowground Tropical Fungal Communities. Microb. Ecol..

[42] Kivlin S.N., Winston G.C., Goulden M.L., Treseder K.K. (2014). Environmental filtering affects soil fungal community composition more than dispersal limitation at regional scales. Fungal Ecol..

[43] Fuhrman J.A. (2009). Microbial community structure and its functional implications. Nature.

[44] Ding J.J., Zhang Y.G., Deng Y., Cong J., Lu H., Sun X., Yang C.Y., Yuan T., Van Nostrand J.D., Li D.Q. (2015). Integrated metagenomics and network analysis of soil microbial community of the forest timberline. Sci. Rep..

[45] Stouffer D.B., Bascompte J. (2011). Compartmentalization increases food-web persistence. Proc. Natl. Acad. Sci. USA.

[46] Montoya J.M., Pimm S.L., Sole R.V. (2006). Ecological networks and their fragility. Nature.

[47] Berry D., Widder S. (2014). Deciphering microbial interactions and detecting keystone species with co-occurrence networks. Front. Microbiol..

