# Supplementary material for: Contrasting Patterns and Drivers of Soil Fungal Communities between Two Ecosystems Divided by the Treeline
Source: Microorganisms. 2021 Nov 2;9(11):2280. doi: 10.3390/microorganisms9112280 (PMC8622436; doi:10.3390/microorganisms9112280)
Supplement: Supplementary file 1 [file microorganisms-09-02280-s001.zip › microorganisms-1424207-supplementary.pdf]

microorganisms

Table S1. Summary of fungal community composition between forest and meadow and significance of differences based on *t*-tests.

	Forest (%)	Meadow (%)	*t* Tests (*p* Value)
**phylum**			
Ascomycota	55.08 ± 19.05	67.29 ± 16.33	**0.031**
Basidiomycota	29.65 ± 21.38	8.51 ±6.6	**0.001**
Chytridiomycota	0.05 ± 0.12	0.1 ± 0.11	0.267
Glomeromycota	0.24 ± 0.54	0.66 ± 1.11	0.127
Zygomycota	3.8 ± 0.85	4.12 ± 0.73	0.286
**Trophic Mode**			
Saprotroph	24.04 ± 10.84	25.99 ± 10.36	0.554
Symbiotroph	29.17 ± 20.89	12.07 ± 8.01	**0.001**
Pathogen	4.03 ± 3.01	3.6 ± 2.57	0.621

Table S2. Spearman's correlation between relative abundance of functional guilds and environmental variables

r-Value	Animal.Pathogen	Arbuscular.Mycorrhizal	Dung.Saprotroph	Ectomycorrhizal	Endophyte	Ericoid.Mycorrhizal	Fungal.Parasite	Leaf.Saprotroph	Lichenized	Orchid.Mycorrhizal	Plant.Saprotroph	Soil.Saprotroph	Undefined.Saprotroph	Wood.Saprotroph
herbrich	0.01	0.38	0.25	**−0.57**	0.02	−0.17	−0.03	**0.55**	0.02	0.44	0.45	−0.12	−0.14	−0.34
plantrich	−0.07	0.04	0.00	−0.09	0.05	0.12	0.20	0.22	−0.03	0.20	0.42	−0.03	−0.15	−0.07
ST	−0.03	−0.15	−0.36	**0.49**	−0.16	0.39	0.12	−0.43	−0.14	−0.30	−0.15	−0.04	−0.02	0.13
SM	0.29	−0.03	0.00	0.02	0.03	−0.31	0.02	−0.01	0.19	0.18	−0.22	0.03	−0.16	0.11
litterthick	−0.04	−0.03	−0.23	0.17	−0.25	0.05	−0.15	−0.33	0.07	0.03	−0.01	0.03	−0.09	0.08
BD	−0.32	0.02	−0.14	0.06	0.10	0.47	−0.12	0.11	−0.05	−0.11	0.13	0.12	−0.04	0.02
pH	0.06	−0.19	0.37	−0.28	−0.08	−0.26	0.17	0.23	−0.41	−0.12	0.27	0.06	0.05	−0.03
clay	−0.03	0.04	−0.02	0.00	0.27	0.28	0.14	−0.06	0.03	0.00	0.14	0.09	0.19	0.13
silt	−0.08	0.37	0.16	−0.46	−0.27	−0.29	−0.22	0.39	−0.10	0.27	0.20	−0.14	−0.12	−0.23
sand	0.14	−0.34	−0.14	0.38	−0.02	0.02	0.08	−0.25	0.08	−0.19	−0.27	0.03	−0.08	0.14
TN	0.13	0.21	0.34	−0.41	−0.05	**−0.61**	−0.27	0.27	0.11	0.15	0.15	0.09	−0.03	−0.10
TC	0.14	0.13	0.35	−0.35	−0.01	**−0.60**	−0.21	0.20	0.13	0.17	0.13	0.14	0.04	−0.08
C:Nratio	0.11	−0.23	0.14	0.20	0.18	0.09	0.32	−0.06	0.19	0.16	−0.01	0.00	0.32	0.10
AP	−0.12	0.24	0.30	−0.28	−0.18	−0.34	−0.30	0.25	0.15	0.07	0.09	−0.19	−0.03	−0.11
AN	0.16	0.19	0.34	−0.38	−0.02	**−0.52**	−0.23	0.27	0.11	0.17	0.16	0.06	−0.04	−0.11

$P < 0.05$ is shown in bold. SM, soil moisture; TN, total nitrogen; TC, total carbon; C:N ratio, ratio of total carbon and total nitrogen; AP, available phosphorus; AN, available nitrogen; TP, total phosphorus.

Table S3. Molecular ecological network and random network parameters of forest and meadow soil fungal communities

Location	Similarity Threshold	Nodes	Links	Molecular Ecological Network					Random Network		
				Average Connectivity	Average Geodesic Distance	Average Clustering Coefficient	Modularity	r^2	Average Geodesic Distance	Average Clustering Coefficient	Modularity
Forest	0.60	69	122	3.56 *	4.63 *	0.21	0.56 *	0.82	3.351 ± 0.105	0.064 ± 0.020	0.464 ± 0.014
Meadow	0.60	91	252	5.54 *	3.01 *	0.23	0.46 *	0.75	2.796 ± 0.039	0.099 ± 0.017	0.344 ± 0.012

*, $P < 0.05$, representing significant differences between the molecular ecological networks of forest and meadow soil fungal communities.

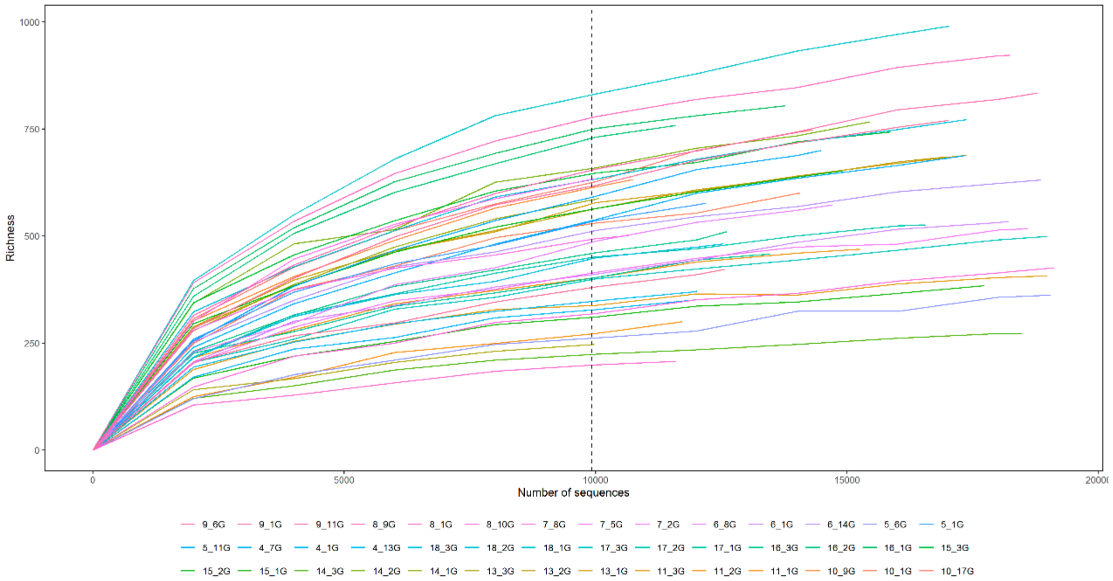

Figure S1. Rarefaction curves of high-throughput sequencing data of the samples.

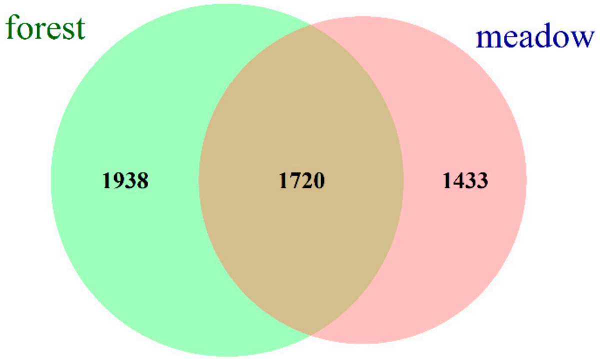

Figure S2. Venn diagram showing numbers of unique and shared fungal OTUs between forest and meadow.

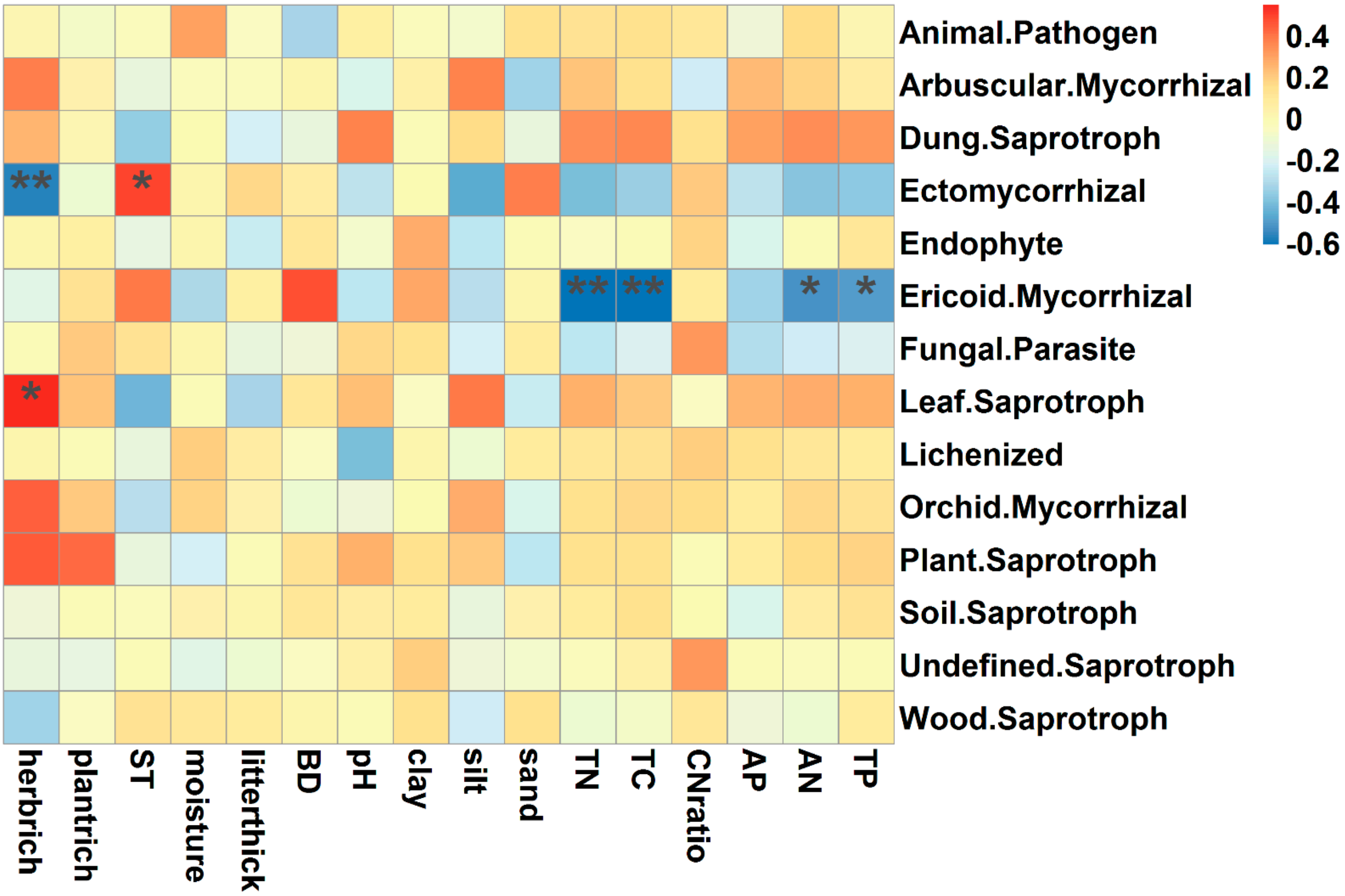

Figure S3. Spearman's correlation between relative abundance of functional guilds and environmental variables. ST, soil temperature; TN, total nitrogen; TC, total carbon; C:N ratio, ratio of total carbon and total nitrogen; AP, available phosphorus; AN, available nitrogen; TP, total phosphorus.
